# Cyclic GMP generated by natriuretic peptide receptor B enhances β_1_-adrenoceptor signaling in normal and failing hearts

**DOI:** 10.1186/1471-2210-11-S1-P46

**Published:** 2011-08-01

**Authors:** Silja Meier, Jan Magnus Aronsen, Ivar Sjaastad, Finn Olav Levy, Tor Skomedal, Jan-Bjørn Osnes, Eirik Qvigstad

**Affiliations:** 1 Department of Pharmacology, University of Oslo and Oslo University Hospital, Oslo, Norway; 2 Center for Heart Failure Research, University of Oslo, Oslo, Norway; 3 Institute for Experimental Medical Research, University of Oslo and Oslo University Hospital, Oslo, Norway

## Background

Atrial (ANP), B-type (BNP) and C-type (CNP) natriuretic peptide levels are increased in heart failure. Natriuretic peptides mediate their effects through natriuretic peptide receptors (NPRs), ANP and BNP preferentially through NPR-A and CNP through NPR-B. NPRs are membrane bound guanylyl cyclases that increase cyclic GMP (cGMP) production when activated. Increased cGMP levels may have beneficial cardiovascular effects through protein kinase G. In contrast, we have previously shown that NPR-B stimulation by CNP enhances β_1_-adrenoceptor (β_1_-AR) mediated signaling in failing hearts through inhibition of phosphodiesterase 3 (PDE3) [1]. This cardioexcitatory influence is longstanding and is thus probably detrimental in the failing heart. However, a comparison of the PDE3 inhibitory effect of NPR-B signaling in non-failing and failing hearts was not elucidated.

## Methods

Heart failure was induced in male Wistar rats by coronary artery ligation. Contraction studies were performed *ex vivo* in left ventricular muscle strips in the presence of appropriate receptor antagonists.

## Results and conclusion

We now demonstrate that CNP through NPR-B is able to potently inhibit PDE3 and thus increase cAMP signaling in non-failing as in failing hearts. This was evident from a marked ability of CNP to potentiate the inotropic and lusitropic responses to β_1_-AR stimulation in left ventricular strips. This conserved mechanism may enhance detrimental β_1_-AR effects during pathological remodeling, and may contribute to the development of the failing cardiac phenotype.
